# Liquiritigenin Ameliorates Metabolic Dysfunction‐Associated Steatotic Liver Disease via AMPK‐Mediated Dual Activation of PPARα/PPARγ

**DOI:** 10.1002/fsn3.72578

**Published:** 2026-11-08

**Authors:** Yu Jung Heo, Jieun Park, Nami Lee, Sung‐E Choi, Ja Young Jeon, Seung Jin Han, Dae Jung Kim, Kwan Woo Lee, Hae Jin Kim

**Affiliations:** ^1^ Institute of Medical Science Ajou University School of Medicine Suwon Republic of Korea; ^2^ Department of Endocrinology and Metabolism Ajou University School of Medicine Suwon Republic of Korea

**Keywords:** AMPK, liquiritigenin, MASLD, PPARα, PPARγ, steatosis

## Abstract

Metabolic dysfunction‐associated steatotic liver disease (MASLD) is characterized by hepatic triglyceride accumulation and chronic inflammation resulting from impaired fatty acid (FA) oxidation and dysregulated metabolic signaling. Liquiritigenin (LQ) exhibits diverse pharmacological activities; however, its role in MASLD and the underlying mechanisms remain unclear. This study aimed to evaluate the therapeutic effects of LQ against MASLD and elucidate its molecular mechanisms, focusing on PPARα/PPARγ activation and AMPK signaling. The effects of LQ were examined in a high‐fat diet (HFD)‐induced MASLD mouse model and in free fatty acid (FFA)‐treated HepG2 cells. Male C57BL/6J mice were fed an HFD for 14 weeks, and LQ was administered during the final 2 weeks. Metabolic parameters, hepatic lipid accumulation, and signaling pathways were analyzed using biochemical and molecular assays and glucose and insulin tolerance tests. LQ markedly reduced hepatic triglyceride content and serum AST and ALT levels in HFD‐fed mice and improved glucose intolerance and insulin resistance. LQ increased AMPK phosphorylation and the expression of SIRT1, PGC‐1α, PPARα, and PPARγ while suppressing *SREBF1* expression and upregulating FA oxidation‐related genes. In FFA‐treated HepG2 cells, LQ activated AMPK, improved insulin signaling (p‐AKT and p‐IRS1), enhanced FA oxidation‐related gene expression, and reduced pro‐inflammatory cytokine levels. Pharmacological inhibition of AMPK abolished LQ‐induced metabolic improvements. In conclusion, LQ attenuates MASLD progression by restoring hepatic metabolic homeostasis through AMPK activation and dual induction of PPARα and PPARγ, promoting FA oxidation and improving insulin sensitivity. Hence, LQ could be a potential natural therapeutic candidate for MASLD.

## Graphical abstract

Liquiritigenin attenuates MASLD by activating AMPK and simultaneously inducing PPARα/PPARγ, thereby enhancing fatty acid oxidation, reducing hepatic steatosis and inflammation, and improving insulin sensitivity in HFD‐fed mice and FFA‐treated HepG2 cells.

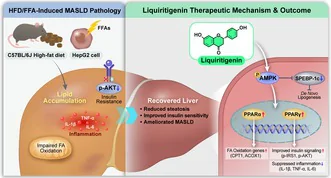

## 1 Introduction

Metabolic dysfunction‐associated steatotic liver disease (MASLD) is a chronic liver disorder characterized by excessive accumulation of triglycerides (TG) within hepatocytes, a pathological condition termed hepatic steatosis (Abdelhameed et al. 2024; Kaylan and Paul 2024). MASLD extends beyond simple lipid deposition and is closely associated with systemic metabolic abnormalities (Riazi et al. 2022). Among the mechanisms implicated in its pathogenesis, impaired fatty acid (FA) oxidation has emerged as a key driver of disease development and progression (Geng et al. 2021). Enhanced lipolysis in adipose tissue increases circulating free fatty acid (FFA) levels, which are subsequently taken up by the liver. When hepatocytes fail to adequately oxidize these FAs, they are esterified into TG and stored (Cheng et al. 2015). This process is further aggravated by increased de novo lipogenesis, mitochondrial dysfunction, and elevated hepatic FFA influx associated with insulin resistance. Collectively, these metabolic disturbances disrupt hepatic energy homeostasis and promote chronic inflammation and fibrosis (Dua et al. 2025). Consequently, MASLD has emerged as a major metabolic disorder wherein defective hepatic FA oxidation constitutes a central pathophysiological link to obesity, Type 2 diabetes, and cardiovascular disease.

Given the multifactorial nature of MASLD, considerable attention has been directed toward natural compounds capable of simultaneously improving metabolic dysregulation and inflammation. Liquiritigenin (LQ) is a bioactive flavonoid derived from licorice (Glycyrrhiza uralensis Fisch., Fabaceae), a plant that is widely used as a food ingredient, natural sweetener, and herbal tea component. Licorice root contains various bioactive constituents, including glycyrrhizin and flavonoids such as LQ and isoliquiritigenin, which contribute to its diverse biological activities (Isbrucker and Burdock 2006). LQ is known to exhibit antioxidant, anti‐inflammatory, and metabolic regulatory activities (Kim et al. 2008; Zhang et al. 2022; Zhou et al. 2022). One proposed mechanism underlying the metabolic effects of LQ involves activation of AMP‐activated protein kinase (AMPK), a central regulator of cellular energy homeostasis (Jung et al. 2017). AMPK activation suppresses anabolic processes such as lipogenesis while promoting the activity of catabolic pathways, including FA β‐oxidation, thereby reducing hepatic lipid accumulation and improving metabolic balance (Hayashi et al. 2000). In addition, the anti‐inflammatory properties of LQ suggest potential benefits in attenuating MASLD‐associated inflammatory responses and improving insulin sensitivity.

Although previous studies have demonstrated metabolic regulatory effects of LQ (Bao et al. 2023; Jung et al. 2017), the mechanistic relationship linking AMPK activation to concurrent improvements in hepatic lipid metabolism, insulin resistance, and inflammation in MASLD has not been fully clarified.

In this study, we hypothesized that LQ ameliorates hepatic steatosis and systemic insulin resistance by promoting FA β‐oxidation through activation of AMPK signaling while suppressing hepatic inflammatory responses. To test this hypothesis, we employed a high‐fat diet (HFD)‐induced MASLD mouse model and an FFA‐treated human hepatocellular carcinoma (HepG2) cell model to investigate the therapeutic effects and molecular mechanisms of LQ. We examined the impact of LQ on hepatic lipid metabolism, AMPK signaling, insulin signaling pathways, and inflammatory responses to evaluate its potential as a natural therapeutic candidate for MASLD.

## 2 Materials and Methods

### 2.1 Animal Study

Eight‐week‐old male C57BL/6J mice (*Mus musculus*) were obtained from Orient Bio Inc. (Seongnam, Republic of Korea). The animals were housed under standard laboratory conditions (22°C ± 2°C; relative humidity 55% ± 10%; 12‐h light/dark cycle) with ad libitum access to food and water. After a 1‐week acclimation period, the mice were randomly assigned to experimental groups. The control group (*n* = 10) received a standard chow diet (control diet; CD), whereas the remaining mice were fed an HFD for 14 weeks to induce MASLD. At Week 12, the HFD‐fed mice were subdivided into groups and administered LQ once daily by oral gavage for 2 weeks at doses of 20 or 40 mg/kg. These doses were selected based on previous studies demonstrating the pharmacological efficacy of LQ and measurable systemic exposure following oral administration within this dose range in rodents (Zhang et al. 2021). LQ (LQ; purity ≥ 98%; Cat. No. HY‐N0377; Lot No. 544036) was purchased from MedChemExpress (MCE, Monmouth Junction, NJ, USA) and suspended in 0.5% methylcellulose. The control and HFD groups received vehicle alone for the same duration. At study termination, the mice were anesthetized and euthanized. Liver tissues were harvested, snap‐frozen in liquid nitrogen, or fixed in formalin for subsequent analyses. Body weight, liver weight, and food intake were also measured (see Supplementary Methods; Supplementary Figure 1).

All animal procedures were conducted in accordance with institutional guidelines and were approved by the Ajou Institutional Animal Care and Use Committee (IACUC approval no. 2022‐0069).

### 2.2 Glucose and Insulin Tolerance Tests

For the glucose tolerance test, mice were fasted for 6 h and intraperitoneally injected with glucose (0.5 g/kg). For the insulin tolerance test, mice received intraperitoneal insulin (0.375 U/kg). Blood samples were collected from the tail vein at 0, 15, 30, 60, 90, and 120 min post‐injection. Blood glucose levels were measured using an Accu‐Chek glucometer (Roche Diagnostics Korea, Seoul, Republic of Korea).

### 2.3 Histological Analysis

Liver tissues were fixed in 4% paraformaldehyde, embedded in paraffin, and sectioned at 5 μm thickness. Sections were stained with hematoxylin and eosin (H&E) for histological evaluation. For neutral lipid detection, liver tissues were embedded in optimal cutting temperature compound, cryosectioned, and stained with Oil Red O (ORO). Slides were digitized and analyzed using 3DHISTECH Slide Converter (SlideMaster, 3DHISTECH Ltd., Budapest, Hungary).

### 2.4 Cell Culture

HepG2 human hepatocellular carcinoma cells (*Homo sapiens*) were obtained from the American Type Culture Collection (ATCC, Manassas, VA, USA). Cells were cultured in Dulbecco's modified Eagle's medium (DMEM; Wellgene, Daegu, Republic of Korea), supplemented with 10% fetal bovine serum (FBS; Gibco, USA) and 1% antibiotic–antimycotic solution (Gibco), and maintained at 37°C in a humidified incubator with 5% CO_2_. At 80%–90% confluence, cells were treated with FFA to induce steatosis, followed by LQ treatment. For AMPK inhibition experiments, cells were pretreated with an AMPK inhibitor Compound C (dorsomorphin; MedChemExpress, Monmouth Junction, NJ, USA) at a 5 μM concentration for 2 h prior to LQ exposure. Cell viability assays using the Cell Counting Kit‐8 (CCK‐8) are described in the Supplementary Methods.

### 2.5 Preparation of FFAs

Palmitic acid (PA; Sigma‐Aldrich, St. Louis, MO, USA) and oleic acid (OA; Sigma‐Aldrich) were dissolved in 0.1 M NaOH at 70°C and conjugated with fatty acid‐free bovine serum albumin (BSA; Sigma‐Aldrich) to generate 10 mM stock solutions. PA and OA were mixed in a 1:2 ratio and diluted in culture medium with appropriate concentration and then administered to cultured cells.

### 2.6 Biochemical Analyses

Plasma TG, aspartate aminotransferase (AST), and alanine aminotransferase (ALT) levels were measured by a central laboratory (SCL Healthcare, Seoul, Republic of Korea). Hepatic TG content was determined using an EZ‐Triglyceride Quantification Assay Kit (DOGEN, Seoul, Republic of Korea) according to the manufacturer's instructions.

### 2.7 siRNA

HepG2 cells were transfected with small interfering RNA (siRNA) targeting PRKAA1 (si AMPK; 5562‐2, Bioneer, Daejeon, Republic of Korea) or non‐targeting control siRNA (SN‐1003, Bioneer, Daejeon, Republic of Korea) at a final concentration of 40 nM using Lipofectamine RNAiMAX Transfection Reagent (Invitrogen, Carlsbad, CA, USA) according to the manufacturer's instructions. The siRNA targeting human PRKAA1 consisted of the following sequences: sense, 5′‐UCC UGG AGA AAG AUG GCG A‐3′; antisense, 5′‐UCG CCA UCU UUC UCC AGG A‐3′. After 48 h of siRNA transfection, cells were treated with FFA with or without LQ for 24 h.

### 2.8 Quantitative Reverse Transcription–Polymerase Chain Reaction (qRT‐PCR)

Total RNA was isolated from tissues or cells using the RNAiso Plus reagent (TaKaRa Bio, Otsu, Japan). Complementary DNA (cDNA) was synthesized using PrimeScript RT Master Mix (TaKaRa Bio, Otsu, Japan). qRT‐PCR was performed with gene‐specific primers designed based on NCBI reference sequences (Table 1) using TB Green Premix Ex Taq (Takara Bio, Otsu, Japan). Primer sequences for additional inflammatory and immune cell markers (Supplementary Figure 2) are provided in the Supplementary Methods.

**TABLE 1:** Primer sequences used for quantitative reverse transcription–PCR.

Gene	Forward primer (5′ → 3′)	Reverse primer (5′ → 3′)
Primers for mouse genes		
*Pparα*	CGAGGTGAAAGATTCGGAAA	GGCCTTGACCTTGTTCATGT
*Acox1*	AACGCTGTGGCTTTGGTGGA	TGGGGACTTCTTGGCCCACT
*Cpt1*	TCGAAACATCTACCATGCAGCA	CAGCATTCTTCGTGACGTTGG
*Cpt2*	GGATTTTGAGAACGGCATTGG	TCATCACGACTGGGTTTGGGT
*Pgc‐1α*	GTCATTCGGGAGCTGGATGG	CAACCAGAGCAGCACACTCT
*Srebf1*	GCTGTTGGCATCCTGCTATC	AGCTGGAAGTGACGGTGGT
*Pparγ*	TTCAGAAGTGCCTTGCTGTG	CCAACAGCTTCTCCTTCTCG
*36B4*	CTGGAGAAACTGCTGCCTCA	TCCACAGACAATGCCAGGAC
Primers for human genes (HepG2 cells)		
*PPARα*	ACCGCAGCTGCAAGATCCAG	TCGTCCAAAACGAATCGCGT
*ACOX1*	ACCCCAAGCTGTACCTTTGA	ATAGGACACCATGCCACACA
*CPT1*	TGGCTTATCGTGGTGGTGGG	GGCCGTTTCCAGAGTCCGAT
*CPT2*	GACCAGTGAGAACCGAGACA	GTCCACTTTCCTCAGGCTCT
*PGC‐1α*	CAAGGCTGGAAGAAGACTGG	GCAAAGAGGCTGGTCTTCAC
*SREBF1*	TTTCTCCCCTGGGCCTGGTA	TGGGAAGCTCTGTGGCAGGA
*PPARγ*	CCCTGCAGGAGCAGAGCAAA	TGATCTCCTGCACAGCCTCCA
*IL‐6*	TGTGTGGGCGGCTACATCT	GCCTTGGTCCAGTTGCCTT
*IL‐1β*	CCTTTGGTCCCTCCCAGGAA	GTCTGCCCAGTTCCCCA
*TNFα*	TGAAAGCATGATCCGGGACG	TGAGGAACAAGCACCGCCTG
*GAPDH*	TATAAATTGAGCCCGCAGCC	CCATGGTGTCTGAGCGATGT

Abbreviations: 36B4, acidic ribosomal phosphoprotein P0 (commonly used housekeeping gene for mouse studies); ACOX1, acyl‐CoA oxidase‐1; CPT1, carnitine palmitoyltransferase‐1; CPT2, carnitine palmitoyltransferase‐2; GAPDH, glyceraldehyde‐3‐phosphate dehydrogenase; IL‐1β, interleukin‐1 beta; IL‐6, interleukin‐6; PGC‐1α, peroxisome proliferator‐activated receptor gamma coactivator‐1 alpha; PPARα, peroxisome proliferator‐activated receptor‐α; PPARγ, peroxisome proliferator‐activated receptor‐γ; SREBF1/Srebf1, sterol regulatory element‐binding transcription factor 1; TNF‐α, tumor necrosis factor‐alpha.

### 2.9 Western Blotting

Protein extracts from tissues or cells were prepared using radio‐immunoprecipitation assay lysis buffer supplemented with protease and phosphatase inhibitors. Protein concentrations were determined using the Pierce BCA Protein Assay Kit (Thermo Fisher Scientific, Waltham, MA, USA). Equal amounts of protein were separated via SDS‐PAGE and transferred onto polyvinylidene fluoride (PVDF) membranes (Merck Millipore Ltd., Billerica, MA, USA). Membranes were blocked with 5% BSA and incubated with primary antibodies overnight at 4°C (Table 2). After washing, the membranes were incubated with horseradish peroxidase‐conjugated secondary antibodies for 1 h at room temperature. Protein bands were visualized using enhanced chemiluminescence reagents (Amersham Pharmacia Biotech, Piscataway, NJ, USA).

**TABLE 2:** Antibodies used for western blot analysis.

Antibody	Manufacturer	Catalog no.	Dilution	Host species
Phospho‐AMPKα (Thr172)	Cell Signaling Technology	#2531	1:1000	Rabbit
AMPKα	Cell Signaling Technology	#2532	1:1000	Rabbit
SIRT1	Cell Signaling Technology	#9475	1:1000	Rabbit
PGC‐1α	Novus Biologicals	NBP1‐04676	1:1000	Rabbit
PPARγ	Cell Signaling Technology	#2435	1:1000	Rabbit
PPARα	GeneTex	GTX101098	1:1000	Rabbit
Phospho‐ACC (Ser79)	Cell Signaling Technology	#3661	1:1000	Rabbit
ACC	Cell Signaling Technology	#3676	1:1000	Rabbit
Phospho‐AKT (Ser473)	Cell Signaling Technology	#9271	1:1000	Rabbit
AKT	Cell Signaling Technology	#9272	1:1000	Rabbit
Phospho‐IRS‐1 (Tyr612)	Abnova	PAB12628	1:1000	Rabbit
IRS‐1	Cell Signaling Technology	#2382	1:1000	Rabbit
GAPDH	Cell Signaling Technology	#2118	1:1000	Rabbit

Abbreviations: ACC, acetyl‐CoA carboxylase; AKT, protein kinase B; AMPK, AMP‐activated protein kinase; GAPDH, glyceraldehyde‐3‐phosphate dehydrogenase; IRS‐1, insulin receptor substrate‐1; PGC‐1α, peroxisome proliferator‐activated receptor gamma coactivator‐1 alpha; PPARα, peroxisome proliferator‐activated receptor‐α; PPARγ, peroxisome proliferator‐activated receptor‐γ; SIRT1, sirtuin‐1.

### 2.10 Statistical Analyses

Statistical analyses were performed using GraphPad Prism software (version 10.4.1; GraphPad Software, San Diego, CA, USA). Differences between the groups were assessed using a one‐way or two‐way analysis of variance (ANOVA) followed by Tukey's multiple comparisons test, as appropriate. Data are presented as mean ± standard deviation (SD), and *p* < 0.05 was considered statistically significant.

## 3 Results

### 3.1 Liquiritigenin Alleviates Hepatic Steatosis and Improves Insulin Resistance in HFD‐Fed Mice

Mice were fed an HFD for 14 weeks, with LQ administered during the final 2 weeks, as illustrated in Figure 1A. Body weight and liver weight did not differ significantly between the HFD‐ and LQ‐treated mice (Supplementary Figure 1), indicating that the beneficial effects of LQ were not attributable to reduced food intake or overall weight loss. To evaluate hepatic lipid accumulation, histological analyses were performed. H&E staining demonstrated that compared with the CD‐fed mice, those fed an HFD developed marked hepatic steatosis characterized by microvesicular lipid accumulation and numerous small lipid droplets. In contrast, mice treated with LQ showed a clear reduction in hepatic lipid vacuolization, indicating attenuation of steatosis. Consistently, ORO staining revealed a marked decrease in the size and distribution of hepatic lipid droplets in LQ‐treated mice (Figure 1B). Biochemical analyses supported these histological findings. HFD feeding significantly increased TG levels in both plasma and liver tissues, whereas LQ administration significantly reduced TG levels (Figure 1C,D). The plasma levels of AST and ALT, indicators of hepatocellular injury, were elevated in HFD‐fed mice but were significantly decreased following LQ treatment (Figure 1E,F). Consistent with these findings, qRT‐PCR analysis revealed that LQ treatment also attenuated HFD‐induced hepatic inflammatory cytokine expression and immune cell infiltration (Supplementary Figure 2). As insulin resistance contributes to hepatic steatosis and dysregulated lipid metabolism (Guerrero et al. 2009), glucose and insulin tolerance tests were performed. Compared with the CD‐fed controls, HFD‐fed mice exhibited impaired glucose tolerance and insulin sensitivity. LQ treatment significantly improved both glucose tolerance (Figure 1G) and insulin sensitivity (Figure 1H), indicating amelioration of HFD‐induced metabolic dysfunction.

**FIGURE 1:**
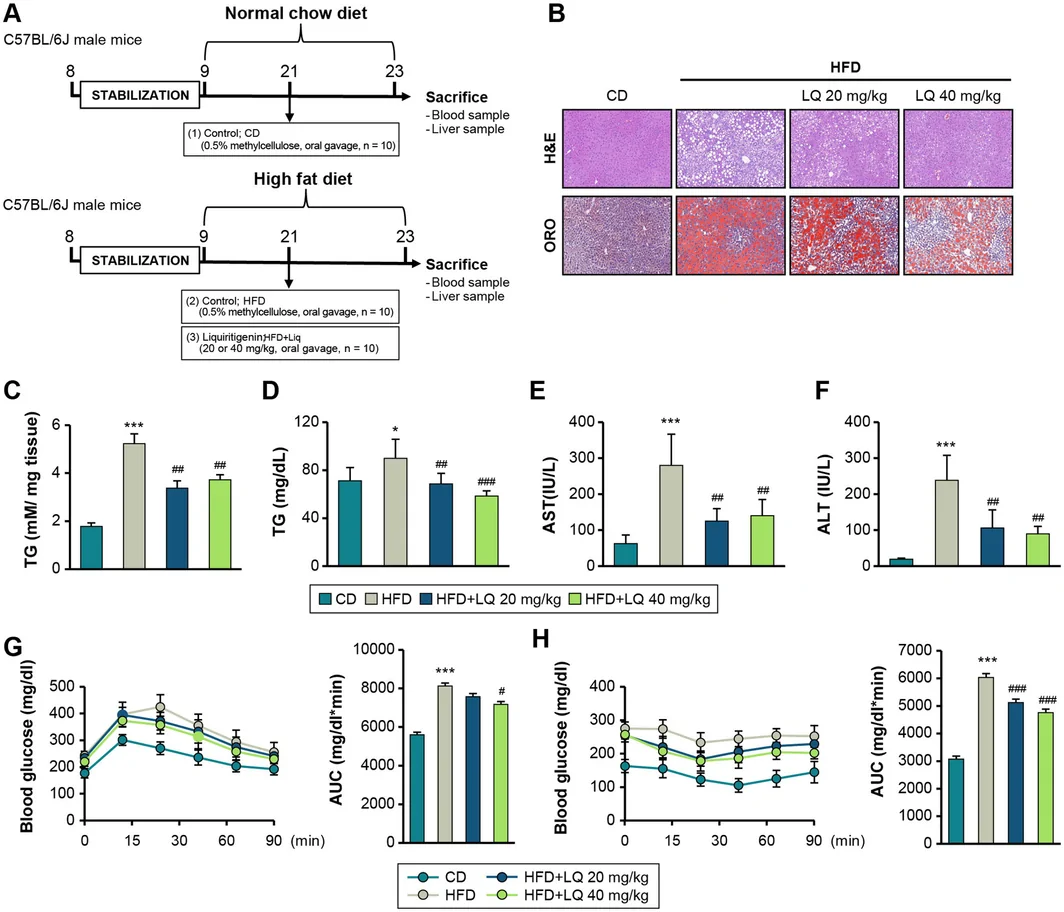
Liquiritigenin attenuates HFD‐induced hepatic steatosis, liver injury, and insulin resistance in mice. (A) Experimental design showing induction of MASLD by HFD feeding for 14 weeks, with LQ administered during the final 2 weeks. (B) Representative H&E and ORO staining of liver sections. Scale bar = 100 μm. (C) Hepatic TG content. (D–F) Plasma TG, AST, and ALT levels (*n* = 10 per group). (G) IPGTT with AUC analysis. (H) IPITT with AUC analysis. Data are presented as mean ± SD (*n* = 10 per group). *p < 0.05, and ***p < 0.001 vs. CD group; #*p* < 0.05, ##*p* < 0.01, and ###*p* < 0.001 vs. HFD group. ALT, alanine aminotransferase; AST, aspartate aminotransferase; AUC, area under the curve; CD, control diet; H&E, hematoxylin and eosin; HFD, high‐fat diet; IPGTT, intraperitoneal glucose tolerance test; IPITT, intraperitoneal insulin tolerance test; LQ, liquiritigenin; MASLD, metabolic dysfunction‐associated steatotic liver disease; ORO, oil red O; SD, standard deviation; TG, triglyceride.

### 3.2 Liquiritigenin Modulates Hepatic Lipid Metabolism in HFD‐Fed Mice

To investigate the mechanism whereby LQ reduces HFD‐induced hepatic lipid accumulation, we examined AMPK, a central regulator of hepatic lipid metabolism that suppresses lipogenesis and promotes FA β‐oxidation. Western blot analysis showed that phosphorylation of AMPK was markedly increased in the livers of HFD‐fed mice treated with LQ compared with that in untreated HFD controls (Figure 2A). We next evaluated the expression of sirtuin‐1 (SIRT1) and its downstream metabolic regulators. LQ treatment increased hepatic protein levels of SIRT1, peroxisome proliferator‐activated receptor gamma coactivator‐1α (PGC‐1α), peroxisome proliferator‐activated receptor‐α (PPAR‐α), and peroxisome proliferator‐activated receptor‐γ (PPAR‐γ) in HFD‐fed mice (Figure 2A,B). Additionally, the RT‐PCR results further confirmed that LQ treatment increased the expression of *Pparα*, *Pparγ*, and *Pgc‐1α* (Figure 2C). These findings suggest that LQ activates key pathways involved in mitochondrial function and lipid oxidation. To further assess whether LQ enhances FA oxidation, we examined the expression of carnitine palmitoyltransferase (*Cpt*)*‐1*, *Cpt‐2*, and acyl‐CoA oxidase‐1 (*Acox1*), both crucial regulators of FA β‐oxidation. The expression of *Cpt1*, *Cpt2*, and *Acox1* was increased by LQ (Figure 2C). The expression of sterol regulatory element‐binding protein‐1 (*Srebf1*), a key transcriptional regulator of lipogenesis (Amemiya‐Kudo et al. 2000), was markedly reduced following LQ treatment. Notably, LQ treatment also increased hepatic *Pparγ* expression (Figure 2B), suggesting coordinated regulation of lipid metabolism and insulin sensitivity.

**FIGURE 2:**
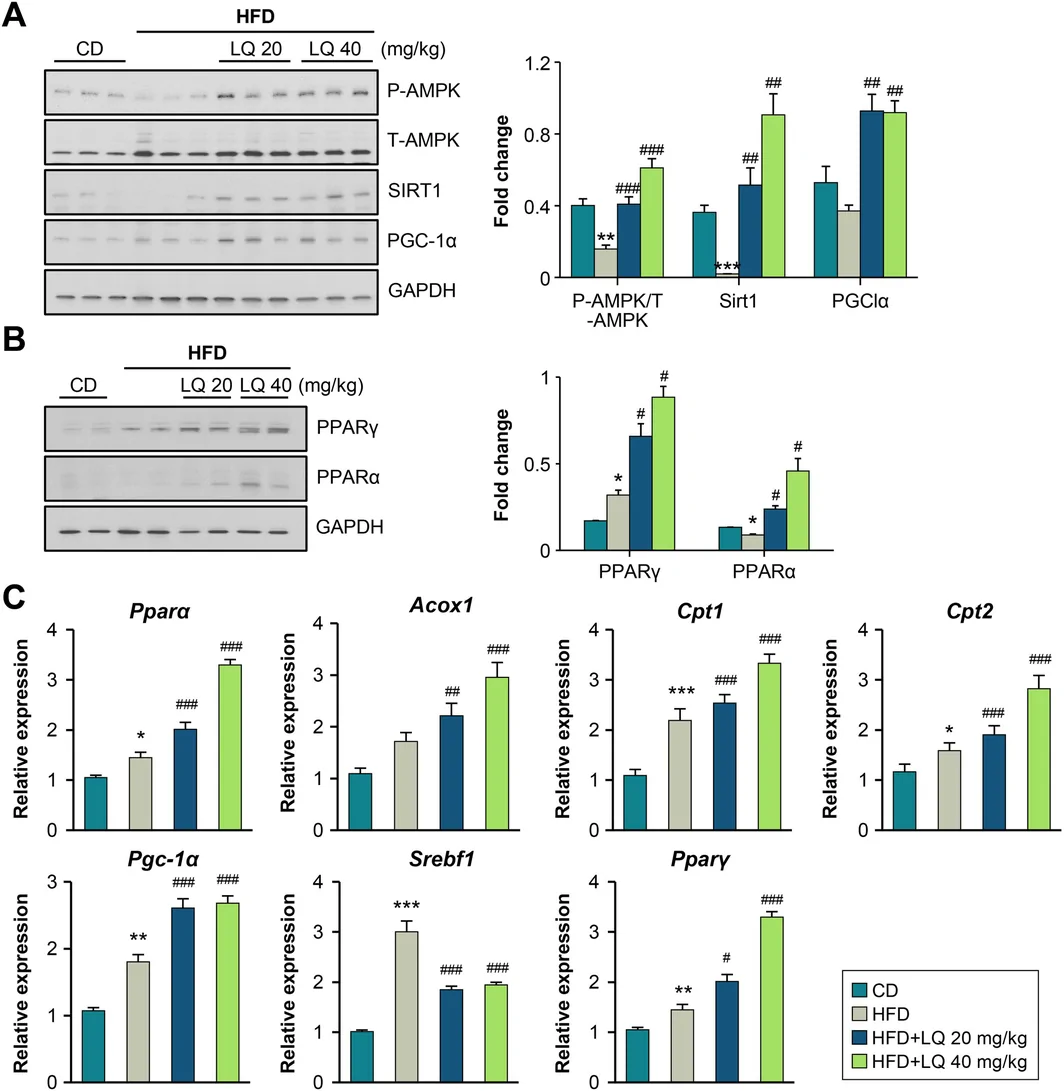
Liquiritigenin activates AMPK signaling and regulates hepatic fatty acid lipid metabolism in HFD‐fed mice. (A) Representative western blot images and quantification of p‐AMPK, total AMPK, SIRT1, and PGC‐1α protein levels in liver tissue. (B) Protein expression levels of PPAR‐γ and PPAR‐α in liver tissue. (C) Relative mRNA expression levels of lipid metabolism‐related genes. Data are presented as mean ± SD. *p < 0.05, **p < 0.01, and ***p < 0.001 vs. CD group; #*p* < 0.05, ##*p* < 0.01, and ###*p* < 0.001 vs. HFD group. *Acox1*, acyl‐CoA oxidase‐1; AMPK, AMP‐activated protein kinase; CD, control diet; *Cpt*, carnitine palmitoyltransferase; HFD, high‐fat diet; LQ, liquiritigenin; p‐AMPK, phosphorylated AMPK; PGC‐1α, peroxisome proliferator‐activated receptor gamma coactivator‐1α; PPARα, peroxisome proliferator‐activated receptor‐α; PPARγ, peroxisome proliferator‐activated receptor‐γ; SD, standard deviation; SIRT1, sirtuin‐1; *Srebf1*, sterol regulatory element‐binding protein‐1.

Collectively, these findings indicate that LQ improves hepatic lipid metabolism in HFD‐fed mice through activation of AMPK signaling and modulation of PPAR‐dependent metabolic pathways.

### 3.3 Liquiritigenin Modulates Lipid Metabolism in Hepatocytes via AMPK Signaling

To further investigate the metabolic regulatory effects of LQ, in vitro experiments were performed using FFA‐treated HepG2 cells to model hepatic steatosis. Cell viability was assessed using the CCK‐8 assay to confirm that LQ at the concentrations used (10–40 μM) did not induce cytotoxicity in FFA‐stimulated HepG2 cells (Supplementary Figure 3). Exposure to FFAs significantly reduced phosphorylation of AMPK, a central metabolic sensor. Treatment with LQ restored phosphorylated AMPK (p‐AMPK) levels in a concentration‐dependent manner and increased the phosphorylation of acetyl‐CoA carboxylase (p‐ACC), a downstream target of AMPK (Figure 3A). Consistent with activation of AMPK signaling, LQ treatment significantly increased the expression of key regulators of FA β‐oxidation, including *PPARα*, *ACOX1*, *CPT1*, and *CPT2*. LQ treatment also increased *PPARγ* expression. In contrast, expression of the lipogenic transcription factor *SREBF1* was markedly reduced following LQ treatment (Figure 3B). To confirm the involvement of AMPK signaling, HepG2 cells were pretreated with Compound C, a pharmacological AMPK inhibitor. Inhibition of AMPK expression significantly attenuated the LQ‐induced phosphorylation of AMPK and reduced the expression of PPARα and PPARγ (Figure 4A). Moreover, the LQ‐induced upregulation of *PPARα*, *ACOX1*, and *CPT2* was markedly suppressed upon AMPK inhibition, whereas the expression of *SREBF1*, which was reduced by LQ, was restored upon AMPK blockade (Figure 4B). To further investigate the AMPK dependency of LQ‐mediated regulation, HepG2 cells were transfected with AMPK siRNA prior to LQ treatment. The efficiency of AMPK knockdown was verified using qPCR (Figure 4C). Knockdown of AMPK markedly attenuated the LQ‐induced increases in PPARα and PPARγ mRNA and protein expression (Figure 4D,E), whereas the LQ‐induced suppression of SREBF1 expression was reversed, restoring its expression to levels comparable to those in the FFA‐treated control group (Figure 4D). These results, consistent with the Compound C inhibition data, further support that AMPK activation is required for the dual regulation of PPARα/γ induction and SREBF1 suppression by LQ.

**FIGURE 3:**
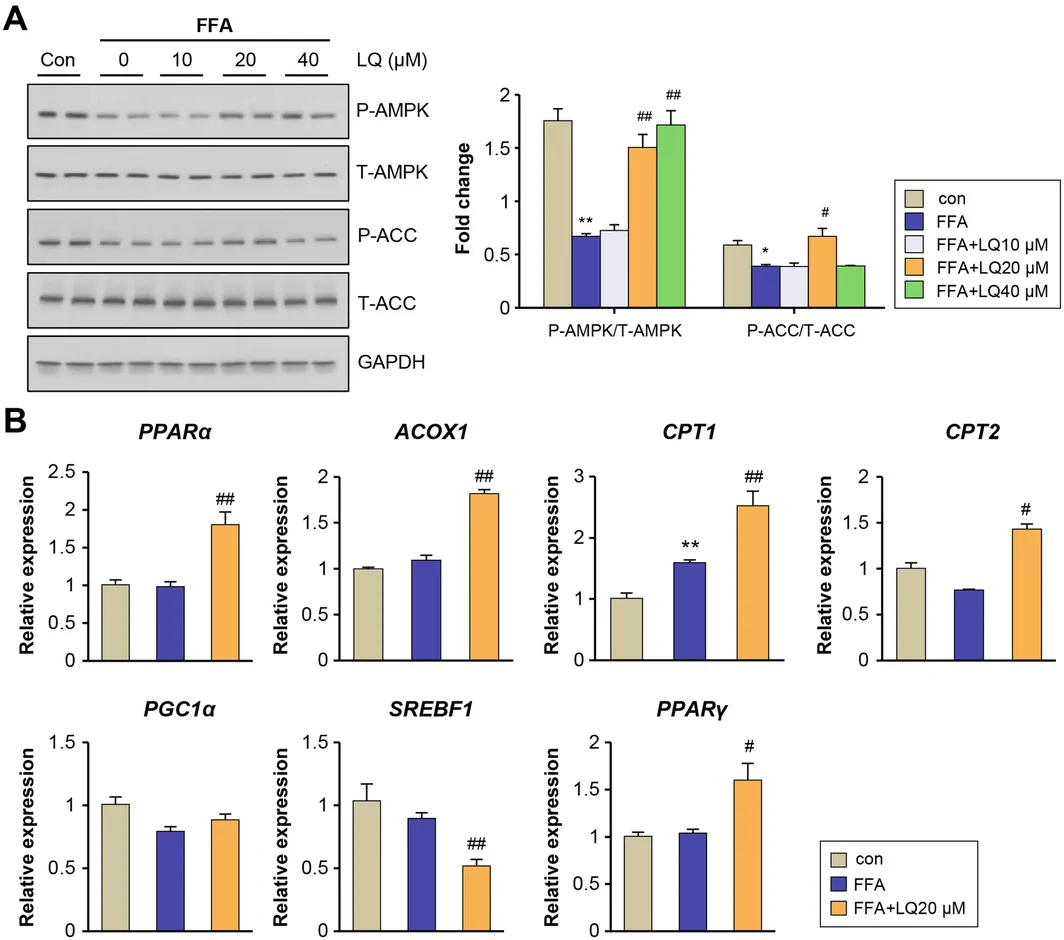
Liquiritigenin activates AMPK signaling and regulates lipid metabolism in FFA‐treated HepG2 cells. HepG2 cells were treated with LQ (0, 10, 20, and 40 μM) for 24 h in the presence of FFAs (400 μM oleic acid and 200 μM palmitic acid). (A) Representative western blot images and quantification of p‐AMPK and p‐ACC. (B) Relative mRNA expression levels of lipid metabolism‐related genes measured via qRT‐PCR. Data are presented as mean ± SD. *p < 0.05 and **p < 0.01 vs. Con group; #*p* < 0.05 and ##*p* < 0.01 vs. FFA group. ACC, acetyl‐CoA carboxylase; *ACOX1*, acyl‐CoA oxidase‐1; AMPK, AMP‐activated protein kinase; Con, control; *CPT*, carnitine palmitoyltransferase; FFA, free fatty acid; LQ, liquiritigenin; *PGC1α*, peroxisome proliferator‐activated receptor gamma coactivator 1α; *PPARα*, peroxisome proliferator‐activated receptor‐α; *PPARγ*, peroxisome proliferator‐activated receptor‐γ; qPCR, quantitative real‐time polymerase chain reaction; SD, standard deviation; *SREBF1*, sterol regulatory element‐binding protein‐1.

**FIGURE 4:**
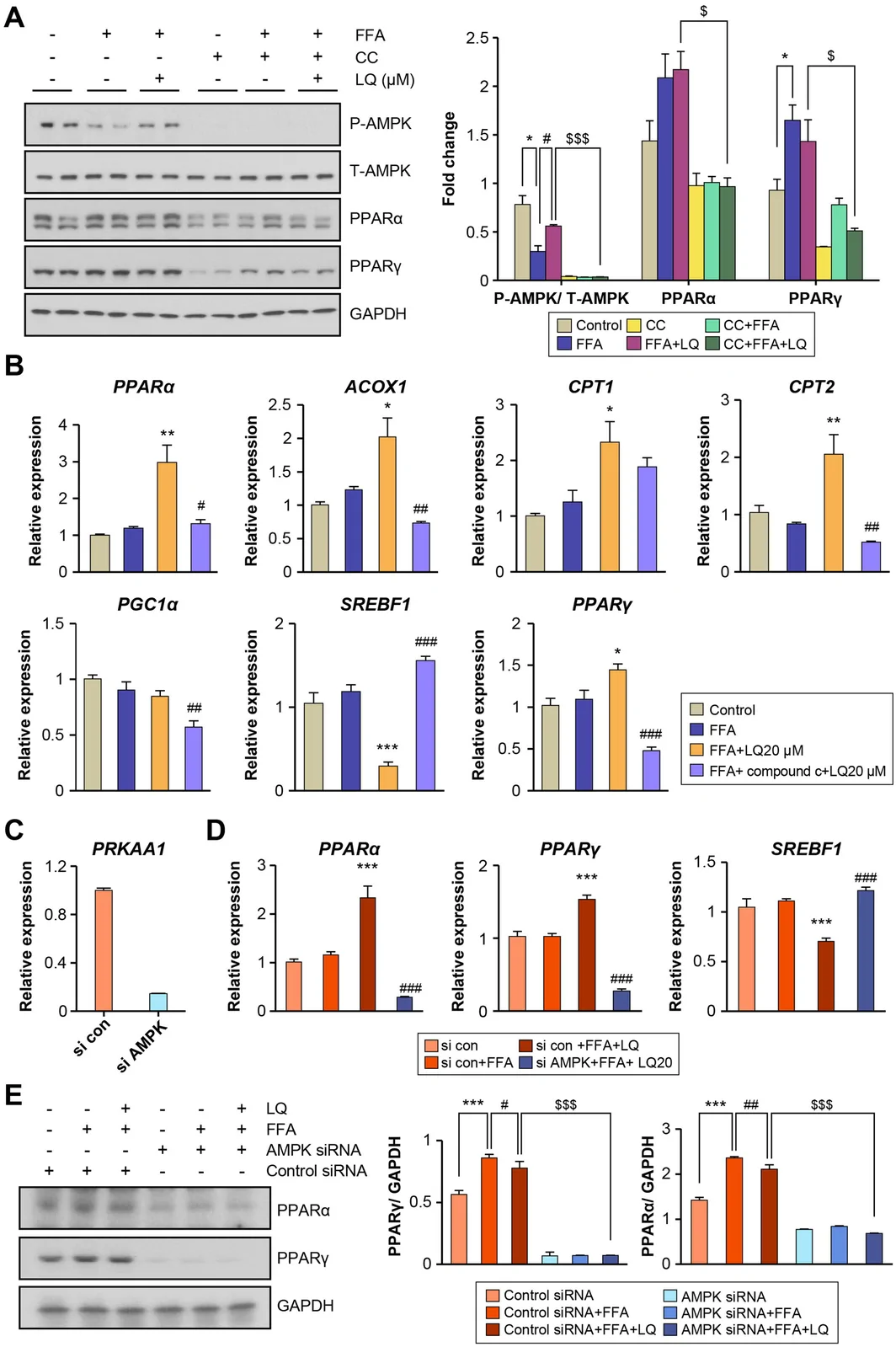
AMPK inhibition attenuates the liquiritigenin‐mediated regulation in FFA‐treated HepG2 cells. HepG2 cells were treated with FFAs and LQ (20 μM) in the presence or absence of Compound C (5 μM), an AMPK inhibitor. (A) Representative western blot analysis and quantification of phosphorylated AMPK, PPARα, and PPARγ protein levels. (B) Relative mRNA expression levels of lipid metabolism‐related genes determined via qRT‐PCR and normalized to *GAPDH* expression. (C) Relative PRKAA1 mRNA expression confirming the efficiency of AMPK knockdown. (D) Relative mRNA expression levels of PPARα, PPARγ, and SREBF1 in control siRNA‐ and AMPK siRNA‐transfected cells under the indicated treatment conditions. (E) Representative western blot analysis and quantification of PPARα and PPARγ protein levels in control siRNA‐ and AMPK siRNA‐transfected cells treated with FFAs and LQ. Data are expressed as the mean ± SD of values from at least three independent experiments. In (A and E), asterisks, hash marks, and dollar signs indicate significant differences between the bracket‐connected groups (*p < 0.05, ***p < 0.001; #*p* < 0.05, ##*p* < 0.01; $$$*p* < 0.001). In (B and D), *p < 0.05, **p < 0.01, ***p < 0.001 vs. FFA (or si con+FFA); #*p* < 0.05, ##*p* < 0.01, ###*p* < 0.001 vs. FFA + LQ (or si con+FFA+LQ). ACOX1, acyl‐CoA oxidase‐1; AMPK, AMP‐activated protein kinase; *CPT*, carnitine palmitoyltransferase; FFA, free fatty acid; GAPDH, glyceraldehyde‐3‐phosphate dehydrogenase; LQ, liquiritigenin; *PGC1α*, peroxisome proliferator‐activated receptor gamma coactivator 1α; PPARα, peroxisome proliferator‐activated receptor‐α; PPARγ, peroxisome proliferator‐activated receptor‐γ; qPCR, quantitative real‐time polymerase chain reaction; SD, standard deviation; *SREBF1*, sterol regulatory element‐binding protein‐1.

Together, these findings indicate that LQ ameliorates FFA‐induced metabolic dysregulation by activating AMPK signaling, thereby promoting FA oxidation and suppressing lipogenesis in hepatocytes.

### 3.4 Liquiritigenin Improves Insulin Signaling and Attenuates Inflammatory Responses in Hepatocytes

To further evaluate the effects of LQ on metabolic dysregulation, its impact on insulin signaling and inflammatory responses was examined in FFA‐treated HepG2 cells. Impaired insulin signaling is a hallmark of lipid‐induced insulin resistance. FFA exposure reduced the phosphorylation of protein kinase B (AKT) and insulin receptor substrate‐1 (IRS‐1), whereas LQ treatment significantly restored the phosphorylation of both proteins, indicating improved insulin responsiveness (Figure 5A). LQ also exerted anti‐inflammatory effects on steatotic hepatocytes. Treatment with LQ significantly decreased the mRNA expression of key pro‐inflammatory cytokines, including interleukin (*IL*)*‐6*, *IL‐1β*, and tumor necrosis factor‐α (*TNF‐α*) (Figure 5B). These findings suggest that LQ improves insulin signaling and suppresses the inflammatory responses associated with lipid‐induced metabolic stress in hepatocytes.

**FIGURE 5:**
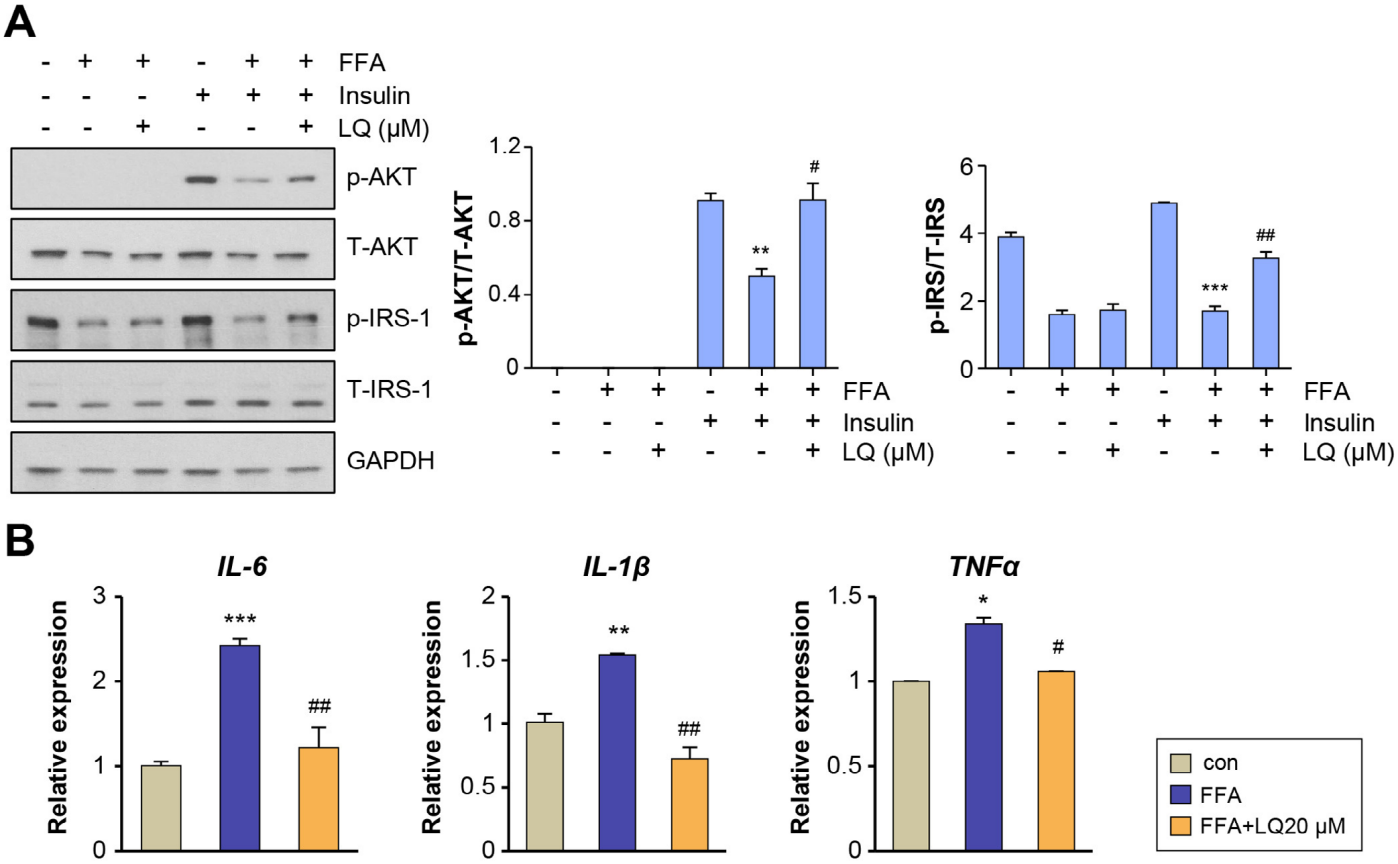
Liquiritigenin restores insulin signaling and suppresses inflammatory responses in FFA‐treated HepG2 cells. HepG2 cells were treated with FFAs and LQ for 24 h, followed by stimulation with insulin (100 nM) for 20 min. (A) Representative western blot images and quantification of p‐AKT and p‐IRS‐1. (B) Relative mRNA expression levels of pro‐inflammatory cytokines measured with qRT‐PCR. Data are presented as the mean ± SD of values from at least three independent experiments. **p < 0.01 and ***p < 0.001 vs. insulin group; #*p* < 0.05 and ##*p* < 0.01 vs. FFA + insulin group (A). *p < 0.05, **p < 0.01, and ***p < 0.001 vs. control group; #*p* < 0.05, and ##*p* < 0.01 vs. FFA group (B). FFA, free fatty acid; *IL‐1β*, interleukin‐1 beta; *IL‐6*, interleukin‐6; LQ, liquiritigenin; p‐AKT, phosphorylated protein kinase B; p‐IRS‐1, phosphorylated insulin receptor substrate‐1; qPCR, quantitative real‐time polymerase chain reaction; SD, standard deviation; *TNF‐α*, tumor necrosis factor‐alpha.

## 4 Discussion

The present study demonstrates the therapeutic potential of LQ against MASLD using both in vivo and in vitro models. LQ markedly attenuated hepatic steatosis, as evidenced by reduced lipid accumulation and decreased plasma and hepatic TG levels, while improving markers of liver injury. These metabolic improvements were accompanied by enhanced glucose tolerance and insulin sensitivity, indicating that LQ ameliorates hepatic steatosis through restoration of systemic metabolic homeostasis. Mechanistically, LQ activated AMPK signaling and concurrently induced both PPARα and PPARγ, thereby enhancing FA oxidation, improving insulin signaling, and suppressing *SREBF1*–mediated lipogenesis. These findings suggest that LQ targets key metabolic pathways underlying MASLD progression rather than merely reducing hepatic lipid accumulation.

Liquiritigenin is a flavonoid naturally present in licorice (Glycyrrhiza spp.), which has long been consumed as a food ingredient—as a flavoring agent in food products (Han et al. 2019) and traditionally regarded as an ancient food and medicinal plant (Zhang et al. 2020). Following oral administration, liquiritigenin is absorbed and can be detected in plasma, supporting its bioavailability as a dietary‐derived compound (Han et al. 2019; Zhang et al. 2020). This food‐relevant background underscores the translational relevance of clarifying how LQ modulates hepatic lipid metabolism in the context of dietary or supplemental exposure to licorice‐derived flavonoids.

AMPK serves as a central regulator of lipid, cholesterol, and glucose metabolism by controlling the phosphorylation of key metabolic enzymes, including ACC, glycogen synthase, hormone‐sensitive lipase, and mechanistic target of rapamycin (mTOR) (Zou et al. 2009). Consistent with this role, hepatic AMPK deficiency exacerbates diet‐induced metabolic dysfunction‐associated steatohepatitis (MASH), highlighting AMPK activation as a promising therapeutic strategy (Alshehade et al. 2022; Zhao et al. 2020). Several natural compounds and traditional herbal medicines have been reported to improve MASLD primarily through AMPK activation, resulting in enhanced FA oxidation and reduced lipogenesis (Fang et al. 2022; Huang et al. 2024; Li et al. 2024). However, the coordinated regulation of both PPARα‐ and PPARγ‐dependent pathways downstream of AMPK has not been fully clarified. In this context, our findings demonstrate that LQ induces the expression of both PPARα and PPARγ, suggesting coordinated modulation of lipid oxidation and insulin‐sensitizing pathways. Activation of PPARα promotes mitochondrial and peroxisomal β‐oxidation, thereby accelerating FA catabolism and reducing hepatic lipid accumulation. In contrast, increased PPARγ expression is associated with improved insulin sensitivity and lipid metabolism in hepatocytes (Hu et al. 2023; Mello et al. 2016; Qiu et al. 2023). Together, these results suggest that LQ‐mediated AMPK activation is associated with the coordinated upregulation of PPARα and PPARγ, accompanied by enhanced regulation of lipid metabolism and improved insulin sensitivity in hepatocytes. Notably, this pattern differs from that of other AMPK activators such as metformin and resveratrol, which are known to suppress PPARα and/or PPARγ activity despite activating AMPK (Sozio et al. 2011; Lin et al. 2012). Contrastingly, LQ‐induced AMPK activation in the present study was accompanied by concurrent upregulation of PPARα and PPARγ and suppression of SREBP‐1c, suggesting that LQ may exert its metabolic effects through coordinated regulation of transcriptional programs involved in fatty acid oxidation and lipogenesis.

This metabolic coordination was further supported by upregulation of the genes involved in FA β‐oxidation. Expression of PPARα and its downstream targets *ACOX1, CPT1*, and *CPT2* was significantly increased in both mouse liver and HepG2 cells. Activation of mitochondrial and peroxisomal β‐oxidation pathways likely contributed to the observed reduction in hepatic lipid accumulation. Conversely, expression of the lipogenic transcription factor *SREBF1* was markedly reduced following LQ treatment, indicating suppression of de novo lipogenesis. In addition, increased expression of SIRT1 and PGC‐1α in vivo suggests enhanced mitochondrial function and biogenesis, further supporting improved lipid oxidation and energy homeostasis. These findings indicate that LQ promotes metabolic reprogramming that enhances lipid utilization while limiting lipid synthesis, thereby restoring hepatic metabolic flexibility.

LQ treatment also improved insulin signaling, a key determinant of MASLD progression. Restoration of phosphorylated AKT and IRS‐1 levels in FFA‐treated hepatocytes indicates that LQ directly alleviates lipotoxicity‐induced insulin resistance. In addition, LQ treatment reduced the expression of pro‐inflammatory cytokines, including *IL‐6*, *IL‐1β*, and *TNF‐α*, consistent with its reported anti‐inflammatory properties (Kim et al. 2008; Yu et al. 2015). Chronic low‐grade inflammation contributes to MASLD progression toward MASH and fibrosis. By suppressing inflammatory mediators while improving insulin signaling, LQ may interrupt the cycle in which lipid accumulation drives inflammation and insulin resistance, thereby exacerbating hepatic dysfunction.

Although hepatic PPARγ activation has been associated with lipogenesis and steatosis under certain pathological conditions, its metabolic functions are highly context‐dependent. In the present study, LQ increased PPARγ expression together with PPARα activation and AMPK phosphorylation, while suppressing SREBP‐1c expression. These findings suggest that the beneficial effects of LQ on hepatic steatosis are primarily mediated by AMPK‐dependent metabolic regulation, including enhanced fatty acid oxidation and reduced lipogenic signaling, rather than by PPARγ‐driven lipogenesis. Additionally, increased PPARγ expression may have contributed to the improvement of insulin resistance and inflammatory responses observed in LQ‐treated mice.

In summary, this study demonstrates that LQ attenuates MASLD progression by restoring hepatic metabolic homeostasis through activation of AMPK signaling. LQ enhances FA oxidation via induction of PPARα and its downstream targets while suppressing *SREBF1*–mediated lipogenesis. Notably, the simultaneous induction of PPARα and PPARγ suggests a dual regulatory mechanism that promotes FA oxidation and improves insulin sensitivity. In addition, LQ alleviated FFA‐induced insulin resistance and inflammatory responses in hepatocytes. These findings support the use of LQ as a promising natural compound for MASLD intervention by targeting key molecular pathways involved in metabolic dysregulation.

## Author Contributions

**Yu Jung Heo:** data curation, formal analysis, funding acquisition, methodology, investigation, writing – original draft. **Jieun Park:** investigation, writing – review and editing. **Nami Lee:** writing – review and editing. **Sung‐E Choi:** writing – review and editing. **Ja Young Jeon:** writing – review and editing. **Seung Jin Han:** writing – review and editing. **Dae Jung Kim:** writing – review and editing. **Kwan Woo Lee:** writing – review and editing. **Hae Jin Kim:** conceptualization, supervision, funding acquisition, writing – original draft, writing – review and editing.

## Funding

This work was supported by the National Research Foundation of Korea (NRF) grant funded by the Korea government (MSIT) (2023R1A2C1004833) and Basic Science Research Program through the National Research Foundation of Korea (NRF) funded by the Ministry of Education (NRF‐2022R1I1A1A01056152).

## Ethics Statement

All animal procedures were conducted in accordance with institutional guidelines and were approved by the Ajou Institutional Animal Care and Use Committee (IACUC approval no. 2022‐0069).

## Conflicts of Interest

The authors declare no conflicts of interest.

## Supporting information


**Supplementary Figure 1.** Effects of liquiritigenin on body and liver weight parameters in HFD‐fed mice.
**Supplementary Figure 2.** Liquiritigenin attenuates hepatic inflammation and immune cell infiltration in HFD‐fed mice.
**Supplementary Figure 3.** Effect of liquiritigenin on cell viability in FFA‐treated HepG2 cells.

## Data Availability

The data used to support the findings of this study are available from the corresponding authors upon reasonable request.
