# Supplementary material for: Liquiritigenin Ameliorates Metabolic Dysfunction‐Associated Steatotic Liver Disease via AMPK‐Mediated Dual Activation of PPARα/PPARγ
Source: Food Sci Nutr. 2026 Nov 8;14(11):e72578. doi: 10.1002/fsn3.72578 (PMC13745806; doi:10.1002/fsn3.72578)
Supplement: Supplementary file 1 [file FSN3-14-e72578-s001.docx]

**Supplementary Information**

**Supplementary Methods**

**Body weight, liver weight, and food intake measurements** Body weight was measured at the time of sacrifice, following 2 weeks of LQ administration. Food intake was expressed as the average daily food intake per mouse (g/day). At the end of the experiment, mice were euthanized, and the liver was excised and weighed immediately. The liver weight-to-body weight ratio was calculated as liver weight (g) divided by final body weight (g).

**Primer sequences for qRT-PCR** The primer sequences used for the genes analyzed in Supplementary Figure 2 were as follows: IL-1β (forward, 5′-TCTCGCAGCAGCACATCAACA-3′; reverse, 5′-CCTG GAAGGTCCACGGGAAA-3′), TNF-α (forward, 5′-TCGTAGCAAACCACCAAGTG-3′; reverse, 5′-AGATAGCAAATCGGCTGACG-3′), IL-6 (forward, 5′-GACCTGTCTATACCACTTCAC-3′; reverse, 5′-GTGCATCATCGTTGTTCATAC-3′), L3T4 (forward, 5′-CCAGCTGTCTGCTTGGATCA-3′; reverse, 5′-AACATGCGTGTGTTCGTGTG-3′), F4/80 (forward, 5′-CAACACTCTCGGAAGCTATT AT-3′; reverse, 5′-GAATTCCTGGAGCACTCATC-3′), CD68 (forward, 5′-AGGGACACTTCGGGCC ATGT-3′; reverse, 5′-GGGTGATGCAGAAGGCGATG-3′), and Ly6G (forward, 5′-TTCCTGCAACA CAACTACC-3′; reverse, 5′-GATGGGAAGGCAGAGATTG-3′).

**Cell viability assay (CCK-8)** Cell viability was assessed using the Cell Counting Kit-8 (CCK-8) assay (Dogen Bio Co., Ltd., Seoul, Republic of Korea). HepG2 cells were seeded in 96-well plates at a density of 5 × 10³ cells/well and treated with FFA in the presence or absence of LQ (10, 20, or 40 μM) for 24 h. After treatment, CCK-8 solution was added to each well and incubated for 2 h at 37°C. Absorbance was measured at 450 nm using a microplate reader, and cell viability was expressed as a percentage relative to the untreated control group.

**Supplementary Figures**

**Supplementary Figure 1. Effects of liquiritigenin on body and liver weight parameters in HFD-fed mice**

Mice were fed a control diet (CD) or high-fat diet (HFD) with or without LQ (20 or 40 mg/kg) for 2 weeks. (A) Liver weight. (B) Body weight. (C) Liver weight-to-body weight ratio. (D) Daily food intake. Data are expressed as the mean ± SD (n = 10 per group). *p < 0.05, ***p < 0.001 vs. CD group.

**Supplementary Figure 2. Liquiritigenin attenuates hepatic inflammation and immune cell infiltration in HFD-fed mice**

Mice were fed a control diet (CD) or high-fat diet (HFD) with or without LQ (20 or 40 mg/kg) for 2 weeks. (A) Relative hepatic mRNA expression of pro-inflammatory cytokines TNFα, IL-6, and IL-1β. (B) Relative hepatic mRNA expression of immune cell markers L3T4 (CD4⁺ T cells), Ly6G (neutrophils), CD68, and F4/80 (macrophages). Data are expressed as the mean ± SD (n = 10 per group). ***p < 0.001 vs. CD group; #p < 0.05, ##p < 0.01, ###p < 0.001 vs. HFD group.

**Supplementary Figure 3. Effect of liquiritigenin on cell viability in FFA-treated HepG2 cells**

HepG2 cells were treated with FFA in the presence or absence of LQ (10, 20, or 40 μM) for 24 h. Cell viability was determined using the CCK-8 assay and expressed as a percentage relative to the untreated control group. Data are expressed as the mean ± SD of values from at least three independent experiments.
